# Imaging equity: Why vascular neurologists need routine ultrasound training

**DOI:** 10.1016/j.neuros.2026.100050

**Published:** 2026-04-21

**Authors:** Ali Zahir, Bruce Ovbiagele

**Affiliations:** Department of Neurology, University of California, San Francisco, USA

**Keywords:** Cerebrovascular disease, Stroke, Ultrasound, Health equity, Disparities, Global health

## Abstract

Stroke disparities persist globally and are driven in part by diagnostic delays and uneven access to advanced neuroimaging. Cerebrovascular ultrasound offers a portable, bedside modality that can optimize stroke evaluation across diverse clinical environments, yet formal training in its use remains inconsistent. This perspective argues that structured, competency-based ultrasound education within vascular neurology fellowships represents a practical, equity-aligned intervention to address diagnostic gaps. While training alone cannot resolve structural drivers of inequity, standardizing ultrasound proficiency can equip clinicians with adaptable diagnostic skills, support timely decision-making, and promote more equitable stroke care delivery across practice settings.

Stroke remains a leading global cause of death and disability. This burden, however, is not distributed equally. Low- and middle-income countries account for a disproportionate share of stroke incidence, mortality, and stroke-associated disability globally [1]. Within the United States, rural and remote populations experience higher stroke incidence and mortality than urban populations, with disparities that have persisted or widened over the past decade despite substantial investments in stroke systems of care [2]. One contributor is diagnostic delay across the stroke care pathway. Although guidelines recommend brain imaging within 25 min of presentation, under-resourced settings and non-comprehensive stroke centers lack timely access to advanced imaging modalities such as CT angiography, perfusion imaging, or MRI [3]. As a result, the benefits of modern stroke therapies may be unevenly realized across populations and geographies.

Ultrasonography offers a rapid, portable bedside diagnostic tool with important implications for equity in stroke care. Transcranial Doppler (TCD) can identify large vessel occlusions, characterize collateral flow patterns, and provide real-time hemodynamic information that complements structural imaging. Combined with clinical assessment, TCD can identify patients who may be eligible for endovascular thrombectomy, including those in rural or remote environments [4]. Carotid ultrasound similarly provides complementary information to angiographic imaging by identifying mobile thrombi, grading carotid occlusion, and distinguishing acute from chronic disease through collateral flow assessment [4]. Together, these modalities illustrate how ultrasound augments diagnostic capacity in resource-constrained settings.

Beyond adult ischemic stroke, cerebrovascular ultrasound has well-established equity-relevant applications in pediatric populations. TCD screening is a cornerstone of primary stroke prevention in children with sickle cell disease (SCD). Elevated cerebral blood flow velocities identify children at high risk for ischemic stroke and guide initiation of chronic transfusion therapy, reducing first-stroke risk by more than 90% [5]. This intervention is particularly salient given the high prevalence of SCD in low- and middle-income countries, where the burden of childhood stroke is substantial and access to advanced neuroimaging may be limited. Despite strong guideline endorsement, implementation of TCD screening remains limited in many regions due to shortages of infrastructure and trained operators.

Despite its expanding clinical utility, there are no uniform training requirements or competency standards specific to cerebrovascular ultrasound within neurology or vascular neurology fellowships in the United States [6]. Surveys of fellowship programs reveal incomplete reporting of ultrasound training components and substantial heterogeneity in educational expectations [7]. In the absence of clear standards, exposure often depends on local expertise, equipment, or informal mentorship, producing wide variability in trainee skill acquisition. Clinicians who complete training without structured exposure may face barriers to incorporating ultrasound into routine practice, particularly in settings where advanced imaging is limited or delayed. Conversely, clinicians trained in environments with robust ultrasound expertise may carry these skills forward, reinforcing geographic and institutional clustering of diagnostic capability.

International differences further highlight the lack of consensus in training paradigms. In several European countries, neurosonology is more formally incorporated into neurology training pathways through structured curricula and competency expectations supported by professional societies. A recent multinational survey identified substantial variability in neurosonology training and certification across Europe, underscoring both the presence of formalized pathways and persistent heterogeneity in implementation [8]. In contrast, U.S. training frequently relies on institution-specific practices or informal apprenticeship models. Together, these observations reinforce the need for portable, standardized training frameworks.

Professional certification pathways in neurosonology exist through national and international organizations, including the American Society of Neuroimaging. These certifications provide mechanisms for quality assurance and professional recognition [9]. However, certification is typically pursued after fellowship and is not uniformly accessible, often depending on local resources, mentorship, and protected time. As a result, reliance on post hoc certification may concentrate expertise among clinicians with greater institutional support rather than democratizing access to diagnostic skills.

Vascular neurology fellowship represents a uniquely formative period during which cerebrovascular physiology, neuroimaging, and time-sensitive clinical decision-making converge. Ultrasound is particularly well suited to this intersection, providing real-time physiologic data that can inform acute management and longitudinal care. When ultrasound skills are acquired informally or deferred until after fellowship, access to guidance, equipment, and supervised practice becomes highly variable, contributing to downstream differences in clinical practice.

Ultrasound training should therefore transition from an ad-hoc model to a competency-based model that is both standardized and adaptable across training environments. Fellowship programs should include instruction in ultrasound physics and interpretation, simulation-based training for probe handling and insonation techniques, and longitudinal supervised scanning integrated into clinical care. Assessment should prioritize image acquisition quality, interpretive accuracy, and appropriate clinical integration, consistent with established practice standards [9]. Standardized documentation of competency would further allow portability of skills across institutions and practice settings.

Achieving this transition will require coordinated collaboration with national professional societies, including the American Heart Association, the American Society of Neuroimaging, and the Society of Vascular and Interventional Neurology, which are well positioned to define competency benchmarks, develop standardized curricula, and support certification pathways. At the same time, implementation of a competency-based ultrasound curriculum presents practical challenges. Many fellowship programs lack dedicated neurosonology laboratories, sufficient case volume, or faculty with formal certification in cerebrovascular ultrasound. To address these barriers, programs may adopt hybrid training models that incorporate simulation-based education, shared regional training resources, and cross-disciplinary collaboration with radiology, cardiology, and emergency medicine. Short-term immersion experiences at high-volume centers may further supplement local training limitations.

In low- and middle-income countries, where access to CT, MRI, and other advanced imaging infrastructure may be limited or cost-prohibitive, standardized ultrasound training may function not only as a complementary diagnostic tool but, in some contexts, as a primary modality for physiologic assessment. As discussed above, transcranial doppler can be used at the bedside to identify large vessel occlusion, allowing clinicians to triage patients for transfer to higher-level centers even in the absence of confirmatory vascular imaging. In rural district hospitals or community clinics without advanced imaging, carotid ultrasound may provide critical information regarding carotid occlusion or high-grade stenosis, informing secondary prevention strategies when angiography is not available.

In regions with established telemedicine infrastructure, these capabilities may be further extended through hub-and-spoke models, in which locally trained providers acquire ultrasound images and waveforms that are transmitted to regional stroke centers for real-time interpretation and clinical guidance. Similar models have been successfully implemented in other domains, such as obstetric and cardiac ultrasound, suggesting feasibility for cerebrovascular applications. In this way, ultrasound training can support earlier diagnostic stratification, facilitate more appropriate triage and transfer decisions, and reduce delays in care in settings where traditional imaging pathways are not readily accessible.

Even in high-resource centers, bedside ultrasound complements rather than replaces advanced imaging. Workflow constraints, patient instability, and competing demands frequently delay definitive imaging. Portable ultrasound enables earlier physiologic assessment, supports more timely clinical decision-making, and improves resource allocation.

Sustainability remains a critical consideration, as implementation will require investment in training, equipment, telecommunication infrastructure, and reimbursement mechanisms to support remote interpretation. Upfront costs may include procurement of portable ultrasound devices, development of training curricula, and telemedicine platforms, while recurrent costs include maintenance, connectivity, and personnel time for acquisition and interpretation.

Potential funding pathways include integration into governmental health initiatives focused on stroke systems of care, particularly those addressing rural–urban disparities. Global health partnerships and academic collaborations may support early implementation through capacity-building efforts, including train-the-trainer models that enable local workforce development and reduce reliance on external expertise. Non-profit organizations and philanthropic funding streams may further support equipment acquisition and pilot programs in underserved regions.

Long-term sustainability will depend on alignment with existing health system priorities and reimbursement structures. In higher-resource settings, incorporation into value-based care models or bundled stroke pathways may incentivize adoption by demonstrating reductions in diagnostic delays and unnecessary transfers. In lower-resource settings, integration into national stroke strategies or essential diagnostics frameworks may facilitate scalability. Embedding ultrasound training within existing medical education and workforce development pipelines may further enhance sustainability by normalizing these competencies within routine clinical practice.

Formal ultrasound training in vascular neurology fellowships represents a practical, equity-aligned intervention to improve stroke care. We summarize our proposed framework in Fig. 1, which highlights the progression from training to system-level impact. As portable ultrasound technology becomes increasingly accessible, failure to standardize training risks widening existing disparities. We recommend that fellowship programs implement competency-based ultrasound curricula, professional societies define training standards, and accrediting bodies formally recognize ultrasound proficiency within vascular neurology education. While training alone cannot resolve structural drivers of stroke disparities, it represents a scalable, workforce-centered intervention that directly targets diagnostic delays at the point of care. Ensuring future vascular neurologists possess these skills reflects a commitment to equitable stroke care.

## Figures and Tables

**Fig. 1.**
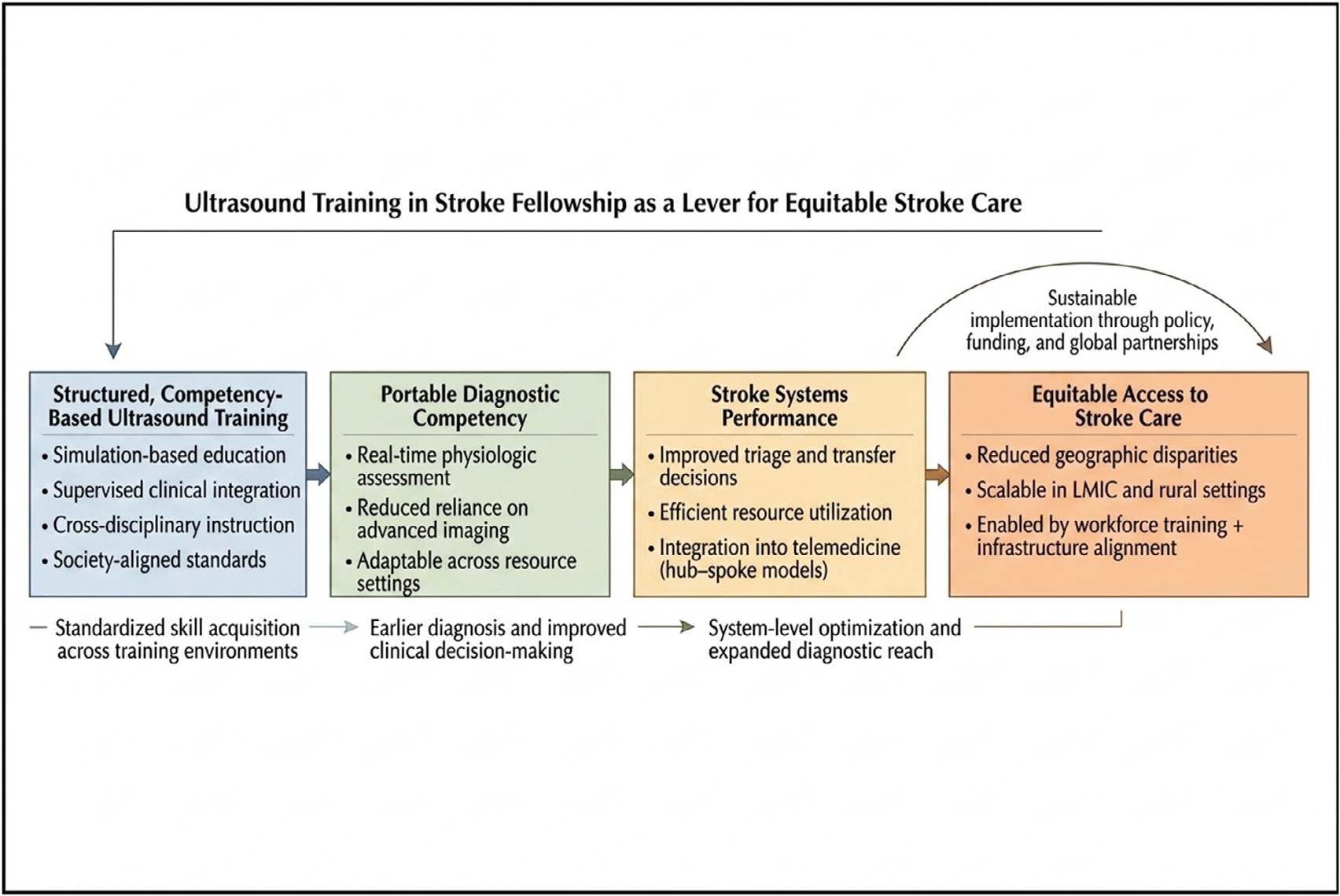
Conceptual framework illustrating how structured ultrasound training during vascular neurology fellowship serves as a leverage point for advancing health equity in stroke care.
